# The predictive role of inflammation indices derived from complete blood count in severe COVID-19 patients: a study from the United Arab Emirates

**DOI:** 10.3389/fmed.2025.1565616

**Published:** 2025-05-14

**Authors:** Rouba Karen Zeidan, Najlaa Al-Bluwi, Hamzah AlZubaidi, Manal Awad, Amal Hussein, Mohamed AlHajjaj, Basema Saddik

**Affiliations:** ^1^Research Institute for Medical and Health Sciences, University of Sharjah, Sharjah, United Arab Emirates; ^2^College of Pharmacy, University of Sharjah, Sharjah, United Arab Emirates; ^3^School of Medicine, Deakin Rural Health, Deakin University Faculty of Health, Warrnambool, VIC, Australia; ^4^Department of Orthodontics, Pediatric and Community Dentistry, College of Dental Medicine, University of Sharjah, Sharjah, United Arab Emirates; ^5^Department of Family and Community Medicine and Behavioral Sciences, College of Medicine, University of Sharjah, Sharjah, United Arab Emirates; ^6^Department of Clinical Sciences, College of Medicine, University of Sharjah, Sharjah, United Arab Emirates; ^7^School of Population Health, University of NSW, Sydney, NSW, Australia

**Keywords:** COVID-19, COVID-19 severity, complete blood count, inflammation ratios, inflammation indices, hematological markers

## Abstract

**Purpose:**

To investigate the predictive effect and determine the cut-off values of complete blood count parameters in severe COVID-19 hospitalized patients in the United Arab Emirates.

**Methods:**

A retrospective observational analytical study analyzed data from 738 medical records of COVID-19 hospitalized patients across several healthcare centers in the United Arab Emirates between 29 January 2020 and 14 October 2021. Complete blood count ratios and indices on admission were evaluated for COVID-19 severity using receiver operating characteristic curves, sensitivity, and specificity.

**Results:**

Main complete blood count-based ratios and indices significantly predicting severe COVID-19 were elevated ratios index (optimal cut-off point ≥3; AOR = 2.8, 95% CI: 1.77–4.42), systemic immune-inflammation index (≥1259.95; AOR = 2.4, 95% CI: 1.53–3.87), systemic inflammation response index (≥3.96; AOR = 2.9, 95% CI: 1.79–4.72), aggregate index of systemic inflammation (≥949.02; AOR = 2.3, 95% CI: 1.43–3.77), platelet-to-lymphocyte ratio (≥188.91; AOR = 2.2, 95% CI: 1.39–3.53), derived neutrophil-to-lymphocyte ratio (≥2.91; AOR = 3.0, 95% CI: 1.84–4.87), and neutrophil-to-lymphocyte ratio (≥6.01; AOR = 3.2, 95% CI: 1.98–5.12).

**Conclusion:**

Identifying hematological markers’ predictive effects and their cut-off values can aid healthcare providers in risk classification and the development of tailored treatment plans. It can also provide cheap, quick, and easy guidance for surveillance systems to lessen the impact of any future outbreaks.

## 1 Introduction

Many people across the world have been relieved by the World Health Organization’s (WHO) announcement on May 5, 2023, that the coronavirus disease 2019 (COVID-19) is no longer causing a pandemic-level threat (1). However, this does not imply that there is no risk to public health from the virus. According to the WHO, people are still dying from this disease worldwide and the number of severe cases admitted to intensive care units (ICU) is relatively high (2). Therefore, it is anticipated that COVID-19 will remain a challenge to the health workforce for many years to come. It will also continue to pose an adverse impact on society, as some individuals still experience short and long-term complications, particularly those who lack access to vaccines or treatments, or who have underlying medical conditions (2, 3).

Nevertheless, it is worth noting that the immediate worldwide response to enhance knowledge, diagnosis, and management of COVID-19 was outstanding. Among the most important COVID-19 topics that have received great attention in public health is hematological alterations. These changes are often unpredictable, rapid, and lethal if not managed immediately (4, 5). Accordingly, complete blood count (CBC) has been proposed as a tool for COVID-19 risk stratification. Its primary advantage lies in being simple, rapid, cost-effective, and universally available. It also provides highly informative insights into the number and types of blood cells, as well as into hematological abnormalities (6–8).

White blood cell (WBC) is a valuable marker in CBC results. They are motile cellular components of the blood that play a major role in the immune system by fighting against infection and disease. WBCs consist of three major types of cells: lymphocytes, neutrophils, and monocytes. While lymphocytes are responsible for the specific recognition of foreign agents and their subsequent removal from the body, neutrophils and monocytes are considered the main phagocytic cells of the body (8, 9). The association between WBCs (lymphocytes, monocytes, and neutrophil) counts with severe COVID-19 outcomes has been highlighted in previous studies (7, 10–12).

Hemoglobin (Hb) and platelets (PLT) are other important markers while reading CBC results. Hb is a component of red blood cells (RBC) that passes oxygen to all living tissues. On the other hand, PLT is a component of blood plasma that is responsible for hemostasis and wound healing (8). Recent evidence indicates that Hb levels (12–14) and PLT count (15) are also associated with COVID-19 severity and mortality.

In addition to WBC, Hb, and PLT, CBC has a variety of derived indices such as neutrophil-to-lymphocyte ratio (NLR), platelet-to-lymphocyte ratio (PLR), monocyte-to-lymphocyte ratio (MLR), derived neutrophil-to-lymphocyte ratio (dNLR), neutrophil-to-lymphocyte and platelet ratio (NLPR), aggregate index of systemic inflammation (AISI), systemic inflammation response index (SIRI), and systemic immune-inflammation index (SII). These indices have been used to assess different aspects of a patient’s health (16, 17) as well as to assist in diagnosis and prognosis of COVID-19 in studies carried out in Saudi Arabia (18), Egypt (19), Iran (20), Turkey (10), Pakistan (7), Indonesia (21), China (22, 23), Nigeria (24), Italy (6, 25), Romania (26) and Mexico (27).

Despite earlier research that showed that CBC parameters and their indices were correlated with COVID-19 severity, the normal ranges for these parameters and indices often vary by region, biological, and racial differences. This is particularly important in the UAE, as it is known for its diverse population. Only one previous study in the UAE focused primarily on the inflammatory biomarkers profile of hospitalized COVID-19 patients (28). However, only the predictive effects of selected CBC markers were assessed. In addition, it was confined to a single center in one emirate (28). Therefore, this study aims to address these gaps by investigating the cut-off values and predictive effect of various CBC parameters from multiple healthcare centers in more than one emirate in the UAE. It is expected that the findings from this study will provide comprehensive, reliable, and generalizable measurements.

## 2 Methods

### 2.1 Study design and setting

A retrospective observational analytical study was conducted in four hospitals in Sharjah and Dubai, UAE. These hospitals included one private, two semi-governmental, and one governmental institute offering a wide range of clinical services, and specialized ICU units. These hospitals were chosen for their capacity, their range of departments, and to ensure diverse representation in the study.

### 2.2 Participants and sample size

This study included adults aged 18 years or older who tested positive for COVID-19 using the Real-time Reverse Transcriptase Polymerase Chain Reaction (RT-PCR) test on nasal and/or pharyngeal swab specimens, and were admitted to one of the four selected hospitals between January 29, 2020, and October 14, 2021. Pregnant women and those unable to provide informed consent were excluded from the study.

**Figure 1:**
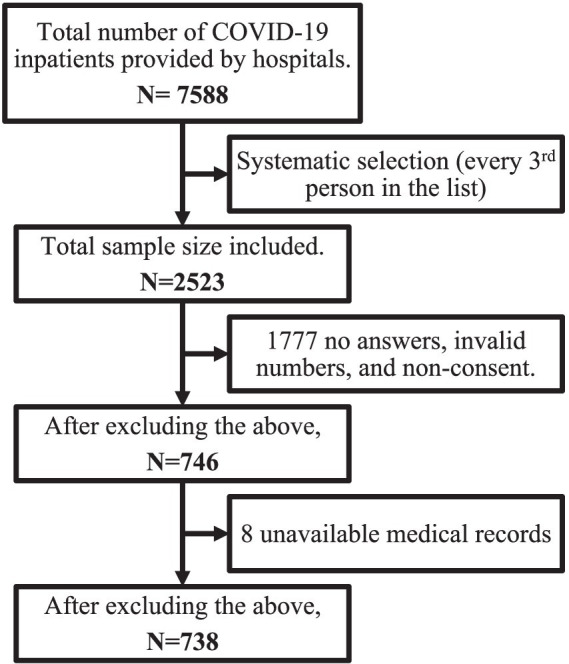
Flowchart of the study sample selection.

Each participating hospital provided a list of inpatients who met the inclusion criteria. Participants were then sorted based on their hospital ID numbers. A systematic random sampling method was then performed where every third person was selected. A minimum of 385 medical records were needed, using the formula of Sample Size = [z^2^ * p(1-p)]/e^2^, assuming a prevalence of 50%, a margin of error of 5%, and a confidence level of 95%. Assuming an attrition and missing data rate of 20%, the target minimum sample size was adjusted to 482 medical records. Figure 1 presents a thorough explanation of how the final sample size was determined.

### 2.3 Data collection and study tools

The study utilized data from medical records of COVID-19 hospitalized patients. Patients or their next of kin were contacted through telephone calls to obtain their consent. For those who consented, medical data were extracted from their electronic health records using a standardized data collection form. This form was a modified version of the WHO/International Severe Acute Respiratory and Emerging Infection Consortium acute respiratory infections (ISARIC) case record form. Data extracted for this study included sociodemographic characteristics (age and gender), vaccination status, symptoms on admission, past medical history, BMI categories (The body mass index of participants were categorized into non-obese if their BMI value was <30 kg/m^2^ and obese if their BMI value was ≥30 kg/m^2^), radiological testing, symptoms, clinical measures, treatments administered, supportive care, complications, and laboratory test results. Any missing or uncertain records were clarified through direct communication with healthcare providers.

Mild cases were defined as those with no oxygen (O_2_) supplementation needed; moderate cases were defined as those requiring O_2_ supplementation or non-invasive ventilation (without ICU admission, intubation, or ECMO); severe/critical cases were those needing ICU admission, intubation, or ECMO.

#### 2.3.1 CBC variables

*Absolute values of CBC:*

This included 6 variables: white blood count (WBC), hemoglobin (Hb), platelet count (PLT), absolute lymphocyte count (ALC), absolute neutrophil count (ANC), and absolute monocyte count (AMC).

*Ratios and indices derived from CBC:*

- Neutrophil-to-lymphocyte ratio (NLR): a ratio calculated by dividing the ANC by the ALC as follows: NLR = ANC/ALC (29).
- Platelet-to-lymphocyte ratio (PLR): a ratio calculated by dividing the PLT by the ALC as follows: PLR = Platelet count/ALC (29).
- Monocyte-to-lymphocyte ratio (MLR): a ratio calculated by dividing the AMC by the ALC as follows: MLR = AMC/ALC (29).
- Derived Neutrophil-to-Lymphocyte Ratio (dNLR): calculated by subtracting the ALC from WBC and then dividing by the ALC as follows: dNLR = (WBC-ALC)/ALC (30).
- Neutrophil-to-Lymphocyte and Platelet Ratio (NLPR): is calculated by multiplying the NLR by 100 and then dividing by the PLT. As follows: (NLR × 100)/ PLT (31).
- Aggregate index of systemic inflammation (AISI): integrates data from ANC, PLT, AMC, and ALC. It is calculated using the formula that follows: AISI = (ANC × PLT × AMC)/ALC (30).
- Systemic Immune-Inflammation Index (SII): combines information from ANC, ALC, and PLT. It is calculated as follows: SII = (Platelet counts× ANC)/ ALC (29).
- Systemic Inflammation Response Index (SIRI): combines information from ANC, AMC, and ALC. The following formula is used to compute it: SIRI = (ANC × AMC)/ALC (30).
- Elevated ratios index (ERI): this index was calculated by adding the number of elevated ratios/indices for each participant. Ratios included were NLR, dNLR, PLR, AISI, SIRI, and SII. Values could range from 0 to 6, with a higher number indicative of greater systemic inflammation.

CBC variables used in this paper were based on laboratory data collected on admission.

### 2.4 Data analysis

Medians and interquartile ranges (IQR) were used to summarize non-normally distributed quantitative variables and frequencies for categorical variables. Normality of distribution of continuous variables was tested visually using Q-Q plots and statistically using Kolmogorov–Smirnov test. Chi-square and Fisher Exact tests were used to analyze frequency comparisons, while Mann–Whitney U test was used to compare medians. Receiver Operating Characteristics (ROC) curves were used to determine the -if CBC ratios and indices could be used as screening tools for COVID-19 severity. Cut-off points were determined using the Youden index. The area under the curve (AUC) and its 95% confidence interval (CI) were calculated. Sensitivity and specificity were also calculated for each parameter. Enter elimination binary logistic regression analysis was utilized to determine the influence of each ratio, index, and ERI on the severity of the disease, after adjusting for all variables that had a *p*-value < 0.05 in the bivariate analysis. Data were analysed using SPSS version 28 (32). A *p*-value < 0.05 (two-tailed) was considered statistically significant.

## 3 Results

### 3.1 Sociodemographic and clinical characteristics

Table 1 presents the demographic and clinical characteristics of 738 hospitalized COVID-19 patients who were categorized into two groups based on disease severity: mild/moderate (*N* = 544, 73.3%) and severe (*N* = 194, 26.3%). Male patients were more likely to have severe COVID-19 compared to female patients (*p* = 0.001), and older age was significantly associated with increased severity (*p* < 0.001). Additionally, patients who had not received their first or second dose of the COVID-19 vaccine (*p* < 0.001) and had a history of diabetes (*p* = 0.003) or hypertension (*p* = 0.014) seemed to also be more severely affected. As for symptoms, fever (*p* < 0.001) and shortness of breath (*p* < 0.001) seemed to be indicative of severe illness.

**Table 1:** Demographic and clinical characteristics of hospitalized COVID-19 patients by disease severity.

Variables		Total		Mild/Moderate		Severe		*p-*value
		*N* = 738		*N* = 544, 73.3%		*N* = 194, 26.3%		
		*N*	%	*N*	%	*N*	%	
Gender	Male	473	64.2%	330	69.8%	143	30.2%	0.001
	Female	264	35.8%	213	80.7%	51	19.3%	
Age (in years)*		50.1 (39.8–63.0)		48.3 (38–62.0)		57.1 (42.3–64.4)		<0.001
Body mass index	Normal weight/Overweight	380	61.5%	291	76.6%	89	23.4%	0.06
	Obese	238	38.5%	166	69.7%	72	30.3%	
COVID first dose	No	411	59.2%	280	68.1%	131	31.9%	<0.001
	Yes	283	40.8%	230	81.3%	53	18.7%	
COVID second dose	No	425	61.2%	291	68.5%	134	31.5%	<0.001
	Yes	269	38.8%	219	81.4%	50	18.6%	
*Comorbidities*								
Hypertension	No	458	62.1%	355	77.5%	103	22.5%	0.003
	Yes	280	37.9%	189	67.5%	91	32.5%	
Diabetes	No	461	62.5%	354	76.8%	107	23.2%	0.014
	Yes	277	37.5%	190	68.6%	87	31.4%	
*Symptoms on admission*								
Fever	No	178	24.1%	150	84.3%	28	15.7%	<0.001
	Yes	560	75.9%	394	70.4%	166	29.6%	
Cough or sore throat	No	187	25.3%	146	78.1%	41	21.9%	0.117
	Yes	551	74.7%	398	72.2%	153	27.8%	
Runny nose	No	711	96.3%	526	74.0%	185	26.0%	0.397
	Yes	27	3.7%	18	66.7%	9	33.3%	
Chest wheezing	No	732	99.2%	542	74.0%	190	26.0%	0.044
	Yes	6	0.8%	2	33.3%	4	66.7%	
Chest pain	No	676	91.6%	492	72.8%	184	27.2%	0.058
	Yes	62	8.4%	52	83.9%	10	16.1%	
Myalgia	No	603	81.7%	432	71.6%	171	28.4%	0.007
	Yes	135	18.3%	112	83.0%	23	17.0%	
Arthralgia	No	715	96.9%	528	73.8%	187	26.2%	0.646
	Yes	23	3.1%	16	69.6%	7	30.4%	
Dizzy and fatigue	No	580	78.6%	419	72.2%	161	27.8%	0.082
	Yes	158	21.4%	125	79.1%	33	20.9%	
Shortness breath	No	311	42.1%	273	87.8%	38	12.2%	<0.001
	Yes	427	57.9%	271	63.5%	156	36.5%	
Headache	No	662	89.7%	479	72.4%	183	27.6%	0.014
	Yes	76	10.3%	65	85.5%	11	14.5%	
Altered consciousness	No	709	96.1%	523	73.8%	186	26.2%	0.871
	Yes	29	3.9%	21	72.4%	8	27.6%	
Abdominal pain	No	687	93.1%	503	73.2%	184	26.8%	0.261
	Yes	51	6.9%	41	80.4%	10	19.6%	
Vomiting or nausea	No	667	90.4%	483	72.4%	184	27.6%	0.014
	Yes	71	9.6%	61	85.9%	10	14.1%	
Diarrhea	No	676	91.6%	493	72.9%	183	27.1%	0.11
	Yes	62	8.4%	51	82.3%	11	17.7%	
Skin rash	No	734	99.5%	540	73.6%	194	26.4%	0.578
	Yes	4	0.5%	4	100.0%	0	0.0%	
Smell and taste	No	725	98.2%	532	73.4%	193	26.6%	0.202
	Yes	13	1.8%	12	92.3%	1	7.7%	
Appetite loss	No	693	93.9%	505	72.9%	188	27.1%	0.042
	Yes	45	6.1%	39	86.7%	6	13.3%	
Urine issues	No	729	98.8%	535	73.4%	194	26.6%	0.122
	Yes	9	1.2%	9	100.0%	0	0.0%	

*Continuous variables are presented as median (Q1–Q3).

### 3.2 Hematological parameters and inflammatory ratios

The comparison of the hematological parameters in severe and non-severe cases revealed significant differences in several parameters. Patients with severe COVID-19 exhibited higher WBC counts [Mild/Moderate: Median (Q1-Q3) = 6.3 (4.8–8.6); Severe: Median (IQR) = 7.8 (5.6–11.2); *p* < 0.001] and ANC [Mild/Moderate: Median (IQR) = 4.6 (3.1–6.4); Severe: Median (IQR) = 5.7 (4.1–9.2); *p* < 0.001], but lower ALC [Mild/Moderate: Median (IQR) = 1.1 (0.8–1.5); Severe: Median (IQR) = 0.9 (0.6–1.2); *p* < 0.001]. All ratios were significantly higher in severe cases. The three inflammation indices also showed significant associations with severity (Table 2).

**Table 2:** Comparison of hematological parameters and inflammatory ratios on admission between patients with mild/moderate and severe COVID-19.

Hematological parameters and inflammatory ratios on admission	Total	Severity		*p*-value
		Mild/Moderate	Severe	
	Median (Q1-Q3)	Median (Q1-Q3)	Median (Q1-Q3)	
WBC count (x 10^9^/L)*	6.6 (5.1–9.2)	6.3 (4.8–8.6)	7.8 (5.6–11.2)	<0.001
Hemoglobin (g/dL)*	13.1 (11.9–14.3)	13.1 (11.9–14.3)	13.1 (11.8–14.4)	0.883
Platelets (x 10 g/L)*	209 (160–277)	209.5 (159–275.5)	208 (164–278)	0.990
Absolute Neutrophil Count (x 10^9^/L)*	4.9 (3.4–7)	4.6 (3.1–6.4)	5.7 (4.1–9.2)	<0.001
Absolute Lymphocytic Count (x 10^9^/L)*	1 (0.7–1.4)	1.1 (0.8–1.5)	0.9 (0.6–1.2)	<0.001
Absolute Monocyte Count (x 10^9^/L)*	0.5 (0.3–0.8)	0.6 (0.4–0.9)	0.5 (0.3–0.8)	0.598
Neutrophil-to-Lymphocyte Ratio (NLR)	4.8 (2.9–8.7)	4 (2.5–7.4)	6.9 (4–11.5)	<0.001
Derived Neutrophil-to-Lymphocyte Ratio (dNLR)	3 (1.9–5)	2.6 (1.7–4.3)	4.1 (2.6–6.3)	<0.001
Platelet-to-Lymphocyte Ratio (PLR)	211 (140.3–316.3)	193.4 (131.2–292.3)	238.7 (167.4–328.9)	<0.001
Monocyte-to-Lymphocyte Ratio (MLR)	0.5 (0.3–0.8)	0.5 (0.3–0.8)	0.6 (0.4–1)	0.013
Neutrophil-to-Lymphocyte and Platelet Ratio (NLPR)	2.3 (1.4–4)	2 (1.2–3.5)	3.1 (1.9–5.8)	<0.001
Aggregate Index of Systemic Inflammation (AISI)	472.3 (225.4–1149.3)	416.1 (216.7–924)	689.3 (261.5–1874.3)	<0.001
Systemic Inflammation Response Index (SIRI)	2.4 (1.2–5.1)	2.2 (1.2–4.1)	3.7 (1.5–7.7)	<0.001
Systemic Immune-Inflammation Index (SII)	971.8 (514.2–1977.5)	827.8 (450–1705.3)	1390 (743.6–2588.3)	<0.001

*Reference values: WBC count (4.0–10.0 × 10^9^/L); Hemoglobin (12.0–15.0 g/dL); Platelets (150–410 × 10^9^/L); Absolute Neutrophil Count (2.0–7.0 × 10^9^/L); Absolute Lymphocytic Count (1.0–3.0 × 10^9^/L); Absolute Monocyte Count (0.2–1.0 × 10^9^/L). References values used by the hospital’s lab.

### 3.3 Optimal cut-off values of hematological parameters and inflammatory ratios

**Figure 2:**
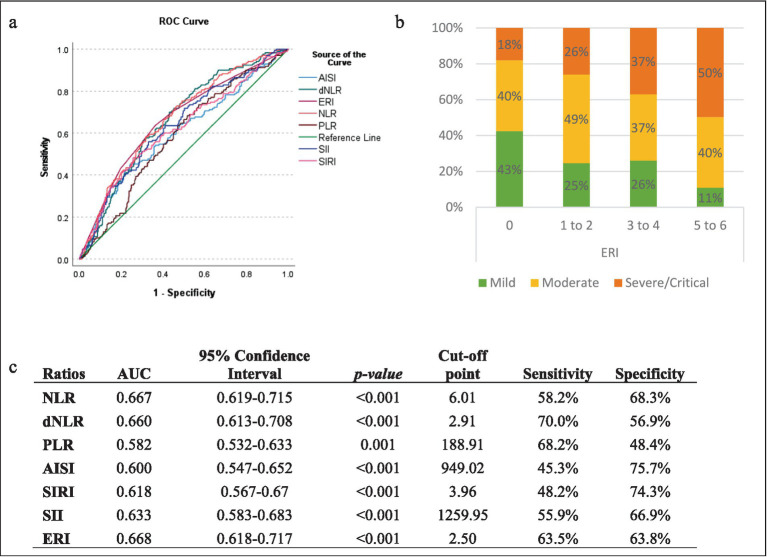
Optimal cut-off points of the different ratios/indices. **(a)** ROC curve analysis of the various indices for distinguishing patients with severe and non-severe COVID-19; **(b)** severity Distribution of COVID-19 patients by elevated ratio index (ERI); **(c)** areas under the curve (AUC) of the ratios for predicting severity in COVID-19 Patients.

The analysis focused on determining optimal cut-off values using ROC analysis. The resulting ROC curves, depicted in Figure 2a, revealed varying areas under the curve (AUC) for the different ratios and indices, all remaining above 0.5, rendering them suitable for further analysis (Figure 2c). The determined optimal cut-off values are presented in Figure 2c, with corresponding specificity and sensitivity values. The ERI showed the highest AUC at a cut-off point of 2.5, indicating that a patient having three or more elevated ratios/indices could indicate a higher risk of developing a severe outcome, with a sensitivity of 63.5% and a specificity of 63.8%, as shown in Figure 2c. The frequency of severe cases was also found to increase gradually with higher ERI (Figure 2b).

### 3.4 Associations between hematological optimal cut-off values and clinical outcomes

To further validate the associations between the various ratios and clinical outcomes, ratios/indices and ERI were dichotomized by their cut-off points to study any association with additional clinical outcomes (death during hospital stay, the number of supportive care measures needed while hospitalized, the number of complications suffered during hospitalization, and the duration of hospital stay). Table 3 delineates these associations, demonstrating significant relationships between the examined ratios and the clinical parameters. Notably, patients with elevated ratios and indices exhibited a substantially higher proportion of severe cases and mortality during hospitalization, accompanied by a higher number of complications and supportive care needed, and a longer hospital stay. Elevated PLR levels (≥ 188.91) were associated with higher rates of severity, supportive care, and number of complications (*p* < 0.001, *p* = <0.001, and *p =* 0.003, respectively), although the association with mortality did not reach statistical significance (*p* = 0.206). Notably, patients with an ERI ≥ 3 exhibited a substantially higher frequency of severe cases (44.5%) and mortality during hospitalization (15.9%) compared to those with an ERI < 3 (21.8 and 6.5%, respectively; both *p* < 0.001). Patients with an ERI ≥ 3 also needed more supportive care measures [2 (1–3) vs. 1 (0–2), *p* < 0.001], had more complications [1 (1–4) vs. 1 (0–2), *p* < 0.001], and had prolonged hospital stays [13 (7–22) vs. 8 (5–15), *p* < 0.001].

**Table 3:** Associations between ratios and clinical outcomes in patients with COVID-19.

Ratios		Severe case			Death during hospital stay			Supportive care procedures needed		Number of complications experienced in-hospital		Days of hospital stay	
		*N*	%	*p*	*N*	%	*p*	Median (Q1-Q3)	*p*	Median (Q1-Q3)	*p*	Median (Q1-Q3)	*p*
NLR^a^	< 6.01	71	21.4%	<0.001	22	6.6%	<0.001	1 (0–2)	<0.001	1 (0–2)	<0.001	8 (5–15)	<0.001
	≥ 6.01	101	45.7%		36	16.4%		2 (1–3)		1 (1–4)		13 (7–23)	
dNLR^b^	< 2.91	51	19.0%	<0.001	14	5.2%	<0.001	1 (0–2)	<0.001	1 (0–2)	<0.001	8 (5–14)	<0.001
	≥ 2.91	120	42.4%		43	15.2%		2 (1–3)		1 (1–4)		12 (7–22)	
PLR^c^	< 188.91	54	22.5%	<0.001	21	8.8%	0.206	1 (0–2)	<0.001	1 (0–2)	0.003	9 (5–17)	0.009
	≥ 188.91	125	38.6%		39	12.1%		1 (1–3)		1 (0–3)		11 (6–19)	
AISI^d^	< 949.02	93	24.5%	<0.001	26	6.9%	<0.001	1 (0–2)	<0.001	1 (0–2)	<0.001	9 (5–16)	<0.001
	≥ 949.02	77	45.6%		31	18.5%		2 (1–3)		2 (1–4)		12 (7–24)	
SIRI^e^	< 3.96	88	23.8%	<0.001	25	6.8%	<0.001	1 (0–2)	<0.001	1 (0–2)	<0.001	9 (5–15)	<0.001
	≥ 3.96	83	45.9%		32	17.8%		2 (1–3)		2 (1–4)		13 (7–24)	
SII^f^	< 1259.95	75	22.9%	<0.001	20	6.1%	<0.001	1 (0–2)	<0.001	1 (0–2)	<0.001	8 (5–15)	<0.001
	≥ 1259.95	96	43.2%		38	17.2%		2 (1–3)		1 (1–4)		12.5 (7–23)	
ERI^g^	< 3	70	21.8%	<0.001	21	6.5%	<0.001	1 (0–2)	<0.001	1 (0–2)	<0.001	8 (5–15)	<0.001
	≥ 3	110	44.5%		39	15.9%		2 (1–3)		1 (1–4)		13 (7–22)	

^a^Neutrophil-to-lymphocyte ratio, ^b^Derived Neutrophil-to-Lymphocyte Ratio, ^c^Platelet-to-lymphocyte ratio, ^d^Aggregate index of systemic inflammation, ^e^Systemic Immune-Inflammation Index, ^f^Systemic Inflammation Response Index, ^g^Elevated Ratio Index.

### 3.5 Hematological ratio predictors of severe COVID-19 patients

**Figure 3:**
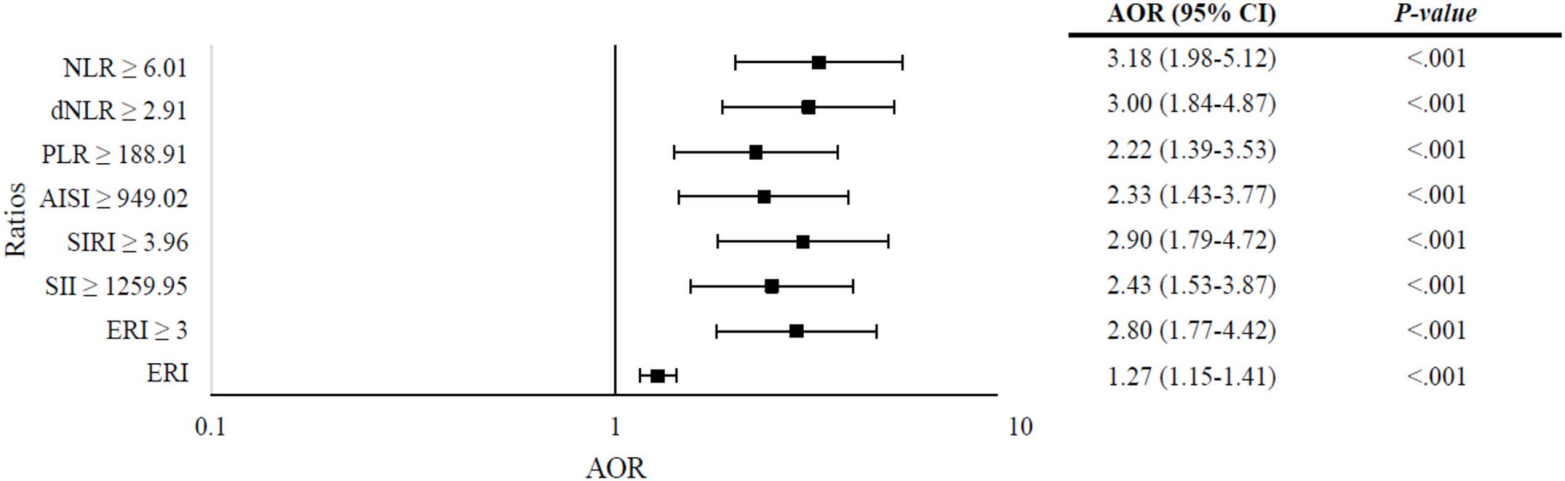
Multivariate logistic regression analysis of ratios predicting severity in COVID-19 patients, adjusted for patient characteristics and symptoms. Models: (a) neutrophil-to-lymphocyte ratio (NLR), (b) derived neutrophil-to-lymphocyte ratio (dNLR), (c) platelet-to-lymphocyte ratio (PLR), (d) aggregate index of systemic inflammation (AISI), (e) systemic immune-inflammation index (SII), (f) systemic inflammation response index (SIRI), (g) elevated ratio index (ERI) ≥ 3; (h) ERI (quantitative). Variables entered in each model: gender, age, obesity, first and second doses of COVID-19 vaccine, hypertension, diabetes, and the following symptoms on admission: fever, myalgia, shortness of breath, headache, and vomiting/nausea. (a) Omnibus test: *p* < 0.001, Nagelkerke R^2^ = 0.241; (b) Omnibus test: *p* < 0.001, Nagelkerke R^2^ = 0.233; (c). Omnibus test: *p* < 0.001, Nagelkerke R^2^ = 0.205; (d) Omnibus test: *p* < 0.001, Nagelkerke R^2^ = 0.206; (e). Omnibus Test: *p* < 0.001, Nagelkerke R^2^ = 0.227; (f) Omnibus test: *p* < 0.001, Nagelkerke R^2^ = 0.215; (g) Omnibus test: *p* < 0.001, Nagelkerke R^2^ = 0.230; (h) Omnibus test: *p* < 0.001, Nagelkerke R^2^ = 0.237.

Figure 3 presents the results of multivariate logistic regression analysis assessing the predictive value of various ratios in determining severity among COVID-19 patients, adjusting for patient characteristics and symptoms. The analysis reveals significant associations between elevated ratios and increased odds of severity. Specifically, an increase in ERI by one unit corresponded to 27% higher odds of severity (AOR = 1.27, 95% CI: 1.15–1.41, *p* < 0.001). Notably, patients with an ERI ≥ 3 exhibited 2.8 times higher odds of severity compared to those with lower ERI values (AOR = 2.8, 95% CI: 1.77–4.42, *p* < 0.001). Similarly, patients with elevated SII (≥ 1259.95) and SIRI (≥ 3.96) demonstrated 2.43-fold (AOR = 2.43, 95% CI: 1.53–3.87, *p* < 0.001) and 2.90-fold (AOR = 2.90, 95% CI: 1.79–4.72, *p* < 0.001) increase in odds of severity, respectively. Additionally, patients with higher values of AISI (≥ 949.02), PLR (≥ 188.91), dNLR (≥ 2.91), and NLR (≥ 6.01) also exhibited significantly increased odds of severity, with AOR ranging from 2.22 to 3.18 and all *p* < 0.001.

## 4 Discussion

In the current study, we examined a group of 738 hospitalized patients who had been diagnosed with COVID-19 in four refereed hospitals in two emirates in the UAE. Our objective was to investigate the predictive effect of hematological indicators among a population from UAE and to establish cut-off points using the results of a simple CBC.

Consistent with previous reports, the increase in WBC, ANC, NLR, and dNLR as well as the decline in ALC, were significantly associated with COVID-19 severity (7, 10, 21, 23–25, 27). One explanation for the harmful effect of increased neutrophil counts is that despite neutrophils being the first cell types to arrive at an infection site to engulf and eradicate harmful microorganisms (9), their persistent production during infection causes them to operate disproportionately, which results in local or systemic damage (33). Conversely, two possible reasons were suggested to explain the decline in lymphocyte count. The first was related to the ability of the virus to directly infect lymphocytes resulting in their death, while the second was linked to the capability of the virus to cause direct damage to lymphatic organs such as the thymus and spleen (34).

Our findings show that the NLR cut-off value for differentiation between severe and non-severe patients was 6.0. This is close to the reported values in Saudi Arabia (5.5) (18) and Indonesia (6.9) (21), but higher than those reported in China (3.3) (23), and Egypt (3.5) (19). Moreover, a cut-off value of 2.9 for dNLR was reported in our study, which is similar to those reported in Egypt (2.9) (19) and China (2.8) (23), but lower than what was reported in Indonesia (4.1) (21). These differences in the cut-off values reported in different countries underscore the importance of determining cut-off values specifically for the UAE population.

The PLR is another calculated CBC ratio. It depends on PLT and ALC and is referred to as a non-specific indicator of inflammation (35). In our population, there was a significantly higher PLR ratio among severe patients with a cut-off value of 188.9. Other studies have shown similar findings but with either lower cut-off values (180.0) (23) or higher cut-off values (192.0) (19) and (295.0) (21). The observed rise in PLR ratio was expected in our participants due to the substantial decrease in lymphocytes relative to the decline in platelet count.

Concerning CBC systemic inflammation indices, the SII is a new index whose calculation is based on platelet, neutrophil, and lymphocyte counts. Severe COVID-19 patients showed a significant increase in the SII ratio in the present study as well in previous studies (24, 25, 27). In which the cut-off point was 1259.95 in our study compared to 812.65, 1260.0, and 2166.0 in Nigeria (24), Italy (25), and Romania (26), respectively. It is worth noting that SII was initially studied in patients with liver tumor (36), lung cancer (37), and myocardial infarction (38) where increased SII ratios were reported in patients with worse prognosis. Such findings were linked to the increased inflammation produced by intense activation of the immune system (25). Later, research on COVID-19 patients showed that COVID-19 produced comparable cellular and immune responses to what was reported in cancer and myocardial infarction patients (35).

Similar to SII, AISI and SIRI are CBC indices that are also used to measure systemic inflammation. AISI and SIRI both reflect the count of neutrophils, lymphocytes, and monocytes. However, AISI also accounts for platelets. The present study demonstrated a significant increase in AISI and SIRI ratios in the severe group compared to the non-severe group with cut-off points of 949.0 for AISI and 4.0 for SIRI. These cut-off points are near to what was previously reported (25, 26). Furthermore, in a meta-analysis, Zinellu et al. (39) described, the predictive role of AISI in discriminating COVID-19 severity independent of the pandemic’s phases or patients’ vaccination status (39). Similarly, SIRI was found to be a predictor of invasive mechanical ventilation and mortality in two studies conducted by Halmaciu et al. (26) and Yılmaz et al. (40).

An interesting finding in our study was that COVID-19 severity was strongly influenced by the presence of multiple elevated ratios/indices (ERI). This is consistent with earlier research conducted in Turkey, which showed that COVID-19 patients with two to three elevated hematological parameters had a higher risk of mortality and ICU admission (41). This finding emphasizes the need to add ERI to risk stratification in addition to other factors that health providers use, such as admission symptoms, comorbidities, and vaccination status. Since even after adjusting for those factors, all the indices remained significantly associated with the severity of disease, mortality, and complications (except for PLR which was not a significant indicator for mortality). Thus, healthcare providers may calculate the number of elevated hematological ratios and indices in the emergency department; if three or more are elevated, the patient should be immediately admitted, closely monitored, and robustly supported.

Given the predictive value of hematological markers in assessing COVID-19 severity, it is important to consider how these markers fluctuated across different waves of the pandemic. Our study period (January 29, 2020–October 14, 2021) encompassed multiple waves of COVID-19 in the UAE: the first wave (March–November 2020), the second wave (November 2020–March 2021), and the third wave (March–July 2021) (42). While our study did not stratify patients by wave, previous research has demonstrated that hematological parameters, including WBC, neutrophils, lymphocytes (43), NLR, dNLR, PLR, SII, and SIRI (44) varied between different waves, with fluctuations in inflammatory markers linked to disease severity and mortality. The studies have reported that inflammatory indices were generally elevated in severe cases and sustained at high levels in fatal outcomes, particularly during earlier waves of the pandemic. The differences in immune response across waves could be attributed to shifts in viral pathogenicity, host immune adaptations, vaccination coverage, and improvements in clinical management strategies (44). The findings of our study, which identified significant elevations in these inflammatory markers as predictors of severe disease, are consistent with the observed trends across different waves. Future studies could explore wave-specific variations in hematological and inflammatory parameters to explore their applicability in the prediction of clinical outcomes.

### 4.1 Strengths and limitations

To our knowledge, this is the first study in the UAE that studied the CBC hematological characteristics of COVID-19 hospitalized patients across several health centers and through multiple phases of the pandemic. The findings underscore the potential utility of CBC ratios and indices as prognostic indicators for adverse clinical outcomes in patients with COVID-19, thereby informing risk stratification and clinical management strategies.

Our study has some limitations. First, 70.4% of the patients on the hospital provided lists either could not be reached or had deactivated lines, which could affect the generalizability of the study findings. It is possible that some patients had to move due to job loss during the pandemic and therefore we could not reach them. Second, it is important to be cautious when generalizing the results to non-hospitalized patients. However, it is noteworthy that our data included medical records of hospitalized patients from the early phase of COVID-19 when even non-severe cases were hospitalized, hence our sample included a relatively high number of mild to moderate cases. Finally, despite our effort to control for potential confounding factors, residual confounding may still exist. For example, inflammation indices are also influenced by cardiovascular diseases and medication used among other factors.

## 5 Conclusion

According to the results of this study, CBC readings of COVID-19 patients obtained at the time of admission including WBC, ANC, ALC, NLR, dNLR, PLR, MLR, NLPR, AISI, SIRI, and SII were significantly associated with the severity of disease in hospitalized patients. CBC markers cut-off points, particular to the UAE population, were established to help healthcare providers in risk classification. In addition, the ERI was found to constitute a good predictive measure with reasonable sensitivity and specificity allowing to reveal patients at a higher risk of developing severe disease. Given the findings of our study as well as the low cost and the ease of use, accurate usage of hematological markers will aid in developing a tailored treatment plan and enable quick delivery of intensive care to individuals who require it most, consequently improving the progression of COVID-19 patients.

## Data Availability

The raw data supporting the conclusions of this article will be made available by the authors, without undue reservation.
